# Long non-coding RNAs as molecular hubs integrating inflammatory and osteogenic pathways in calcific aortic valve disease

**DOI:** 10.3389/fcvm.2026.1843916

**Published:** 2026-07-08

**Authors:** Juan Ignacio Muñoz-Manco, Annisa Mardianing Utami, Zhexi Li, Georg Nickenig, Mohammed Rabiul Hosen

**Affiliations:** 1 Cardiovascular Epigenetics & RNA Biology Group, Molecular Cardiology, Heart Center, Department of Internal Medicine II, University Hospital Bonn, Rheinische Friedrich-Wilhelms University of Bonn Venusberg-Campus 1, Bonn, Germany; 2 Heart Center, Department of Internal Medicine II, University Hospital Bonn, Rheinische Friedrich-Wilhelms University of Bonn Venusberg-Campus 1, Bonn, Germany

**Keywords:** aortic stenosis (AS), biomarker, calcific aortic valve disease (CAVD), long non-coding RNAs (lncRNAs), RNA therapeutics

## Abstract

**Significance:**

Cardiovascular disease remains the leading cause of morbidity and mortality worldwide. Among valvular pathologies, CAVD is the most prevalent and poses a growing burden on the aging population. Once considered a passive degenerative process, aortic stenosis (AS) is now understood to be an actively regulated disease characterized by progressive leaflet fibrosis, calcification, and inflammation, ultimately leading to left ventricular outflow obstruction and heart failure. Current treatment options are limited to surgical or transcatheter valve replacement, as no pharmacological therapies exist to halt or reverse disease progression. This review frames the discussion around the potential of long non-coding RNAs (lncRNAs) as therapeutic targets, rather than implying established therapies.

**Recent advances:**

Through the advancement of genetic manipulation techniques and their application in cardiovascular biology, non-coding RNAs have emerged as dynamic regulators of disease pathogenesis. While initial focus centered on microRNAs, recent evidence highlights lncRNAs as critical modulators of gene expression governing valvular interstitial cell (VIC) biology. LncRNAs influence key pathological processes in AS, including osteogenic differentiation, extracellular matrix remodeling, and inflammatory signaling. Furthermore, circulating lncRNAs, either freely circulating or encapsulated within extracellular vesicles, are emerging as novel mediators of intercellular communication within the valve microenvironment and represent promising candidates for diagnostic and prognostic applications, offering the potential for a *liquid biopsy* approach in AS management. Despite significant advancements in our understanding of non-coding RNA biology, the functional roles of specific lncRNAs in the pathogenesis of aortic stenosis remain largely unexplored. However, emerging evidence from related inflammatory pathways (e.g., NF-κB, MAPK, and JAK/STAT) and other cardiovascular diseases provides a rational basis for investigating the therapeutic potential of lncRNAs in AS, without overstating current knowledge. Elucidating the precise mechanisms by which lncRNAs regulate VIC fate and valvular calcification is crucial for the development of effective targeted interventions aimed at slowing or preventing disease progression and reducing the clinical burden of AS.

**Future directions:**

Key unanswered questions remain: What is the specific lncRNA signature of CAVD? How do individual lncRNAs functionally contribute to disease progression? And how can the delivery and targeting challenges associated with lncRNA-based therapeutics be overcome? This review provides a comprehensive landscape of the current developmental progression of RNA therapeutics, with a specific focus on lncRNA-based strategies as a holistic approach for treating CAVD in preclinical models. Addressing these research priorities will be essential for translating lncRNA-based strategies into clinical applications for this increasingly prevalent disease.

## Introduction

Cardiovascular disease (CVD) remains the foremost cause of mortality and morbidity worldwide, accounting for approximately 19.5 million deaths annually, nearly one-third of all global deaths, with a significant proportion occurring prematurely in individuals under 70 years of age (1, 2). Among the spectrum of CVDs, aortic stenosis (AS) stands as a prevalent and potentially fatal condition, characterized by the progressive narrowing of the aortic valve orifice and consequent obstruction of blood flow from the left ventricle. The most common underlying etiology is calcific aortic valve disease (CAVD), a pathology whose prevalence increases sharply with age, affecting approximately 3%–5% of the population over 75 and conferring a significant mortality risk (1, 3, 4).

Historically considered a passive, degenerative consequence of aging akin to mechanical “wear and tear,” AS is now recognized as an actively regulated and multifactorial disease process (5). Contemporary evidence has redefined AS as a pathology sharing remarkable pathophysiological similarities with atherosclerosis, involving endothelial injury, chronic inflammation, neovascularization, and hemostatic alterations (6–8). These insults trigger a complex cascade of fibrotic remodeling and pathological osteogenic differentiation of valvular interstitial cells (VICs), culminating in progressive leaflet mineralization (7, 8). This maladaptive process is orchestrated by a network of cellular and molecular signals, including infiltrating immune cells, cytokines such as tumor necrosis factor-alpha (TNF-α) and interleukins (IL-1β), lipid mediators, and growth factors like transforming growth factor-beta 1 (TGFβ1). These factors converge on core developmental and osteogenic signaling pathways, including NOTCH, Wnt/β-catenin, and myocardin, ultimately promoting a pro-osteogenic gene program and driving calcium deposition (3, 9).

Despite significant advances in understanding these protein-mediated pathways, the broader regulatory networks controlling them are not fully elucidated. Recently, a new layer of complexity has emerged with the identification of non-coding RNAs (ncRNAs), functional RNA molecules that are not translated into proteins, as critical modulators of gene expression in CVDs (10–12). Among these, long non-coding RNAs (lncRNAs), a class of transcripts longer than 200 nucleotides that do not code for proteins, have gained significant attention (13). LncRNAs regulate gene expression through diverse mechanisms, including chromatin remodeling, transcriptional interference, and post-transcriptional modulation, positioning them as key molecular actors in cardiovascular pathophysiology (13). A compelling example is Metastasis-Associated Lung Adenocarcinoma Transcript 1 (MALAT1), an lncRNA initially characterized in cancer and myogenesis (14, 15), which has since been implicated in CAVD where its upregulation promotes osteogenic differentiation of VICs by sponging the antiosteogenic microRNA, miR-204 (16). LncRNAs are emerging as essential regulatory molecules in the pathogenesis of aortic diseases. Their ability to modulate inflammation, cellular differentiation, and matrix remodeling underscores their potential as both diagnostic biomarkers and novel therapeutic targets. As research continues to elucidate the specific roles of individual lncRNAs in these debilitating conditions, they are likely to provide critical new insights into disease mechanisms and pave the way for innovative therapeutic strategies to mitigate the impact of AS and other aortic diseases (ADs). In this review, we synthesize the current knowledge of lncRNAs in AS, with the objective of highlighting our understanding of their pathobiology, evaluating their clinical relevance, and discussing the challenges associated with the development of emerging lncRNA-based therapeutics.

## The pathobiology of aortic disease: the crossroads of inflammation and mechanics

AS initially follows a prolonged asymptomatic phase during which the left ventricle adapts to progressive pressure overload; however, the eventual onset of symptoms signals a poor prognosis and requires timely intervention (17). The classic symptomatic triad includes exertional dyspnea, resulting from elevated left ventricular filling pressures and diastolic dysfunction; angina, due to increased myocardial oxygen demand from left ventricular hypertrophy (LVH) and reduced coronary perfusion; and syncope, arising from impaired cerebral perfusion during exertion (18). Several systemic conditions accelerate valvular calcification and AS progression, including hyperlipidemia, diabetes mellitus, chronic kidney disease, and inflammatory disorders such as rheumatoid arthritis and systemic lupus erythematosus (19). Patients with congenital bicuspid aortic valve are particularly vulnerable, developing AS earlier because of abnormal hemodynamic flow that promotes rapid calcification, often demanding aortic valve replacement as early as their fifth or sixth decade of life (17–19). As the disease advances, progressive pressure overload produces downstream sequelae, including pulmonary venous congestion, secondary pulmonary hypertension, left atrial dilation with attendant atrial fibrillation, reduced cardiac output, ischemic stroke from calcific emboli, and ultimately heart failure if left untreated (17–20). Critically, AS is not confined to the valve itself; sustained pressure overload induces concentric LVH that initially preserves systolic function but progressively leads to reduced compliance, myocardial fibrosis, and impaired diastolic and systolic performance, culminating in heart failure with preserved or reduced ejection fraction (HFpEF or HFrEF) and conferring an elevated risk of ventricular arrhythmias and sudden cardiac death (14, 15, 21, 22).

The pathogenesis of AS is now recognized as an active and multifactorial process involving endothelial injury, inflammation, neovascularization, alterations in hemostatic and platelet function, and cell signaling cascades driving fibrosis, matrix remodeling, and mineralization (3, 23, 24). As one of the most common chronic and progressive valvular heart diseases worldwide, CAVD is characterized by progressive narrowing of the aortic valve (AV) orifice, which obstructs blood flow from the left ventricle to the aorta and ultimately necessitates concentric LVH and diastolic dysfunction as compensatory responses to sustained pressure overload (17, 18, 24). The disease initiates at the interface of mechanics and biology: mechanical stress and disturbed flow dynamics within the AV induce endothelial injury, promoting the infiltration of oxidized low-density lipoproteins (ox-LDL) into the valve tissue, which, in turn, triggers oxidative stress and a local immune response (12, 24). Similar to atherosclerosis, CAVD is associated with lipid deposition within the valve leaflets, where activated macrophages transform into foam cells upon uptake of excess lipids and secrete proinflammatory cytokines, including interleukin-6 (IL-6), TGF-β, and TNF-α, which perpetuate inflammation, tissue remodeling, and fibrosis (19, 23, 24).

**Figure 1:**
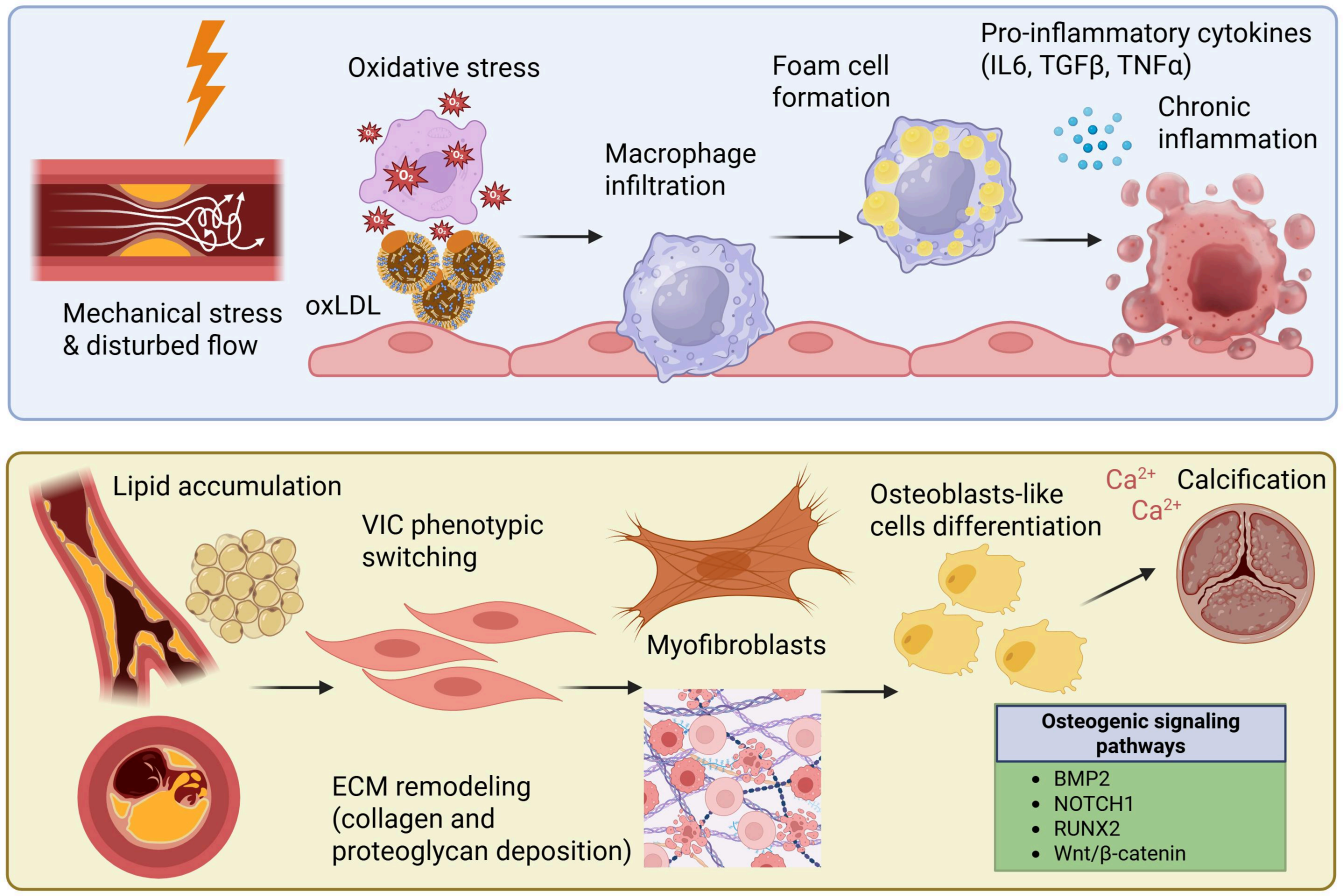
The pathogenic cascade in calcific aortic valve disease (CAVD). A schematic representation of the multifactorial mechanisms driving valve calcification. Mechanical stress and disturbed flow initiate endothelial injury, leading to lipid accumulation, oxidative stress, and macrophage infiltration. Foam cell formation and proinflammatory cytokine secretion (IL-6, TGF-β, TNF-α) establish chronic inflammation and activate valvular interstitial cells (VICs) to undergo phenotypic switching into myofibroblasts and osteoblast-like cells. Myofibroblasts drive extracellular matrix (ECM) remodeling through collagen and proteoglycan deposition, while osteoblast-like cells (regulated by BMP2, NOTCH1, RUNX2, and Wnt/β-catenin signaling pathways) promote calcium deposition (Ca^2+^) and nodule formation. This integrated cascade illustrates the progression from initial injury to advanced fibro-calcific remodeling.

The hallmark of advanced AS is fibro-calcific remodeling of the AV leaflets, driven by the accumulation of ox-LDL and chronic inflammation that together activate VICs to undergo phenotypic transition (6, 24). VICs differentiate into myofibroblasts and osteoblast-like cells, promoting excessive deposition of collagen and proteoglycans that contribute to extracellular matrix (ECM) remodeling and progressive valve stiffening. Over time, calcific deposition replaces fibrotic tissue, further restricting leaflet mobility and exacerbating hemodynamic obstruction (19, 24). This calcification process is molecularly orchestrated by key signaling pathways, including bone morphogenetic protein, neurogenic locus notch homolog protein 1 (NOTCH1), Runt-related transcription factor 2 (RUNX2), and Wnt/β-catenin signaling, which collectively increase the expression of osteogenic genes such as osteocalcin and alkaline phosphatase (ALPL), ultimately driving the formation of calcific nodules within the valve leaflets (Figure 1) (3, 19, 24). At the molecular level, recent transcriptomic studies have implicated ncRNAs, particularly lncRNAs and miRNAs, as critical regulators of valve inflammation, osteogenic differentiation, and extracellular matrix remodeling, highlighting these molecules as promising diagnostic biomarkers and therapeutic targets in AS (25–28).

## Non-coding RNAs in the development of aortic disease pathogenesis

**Figure 2:**
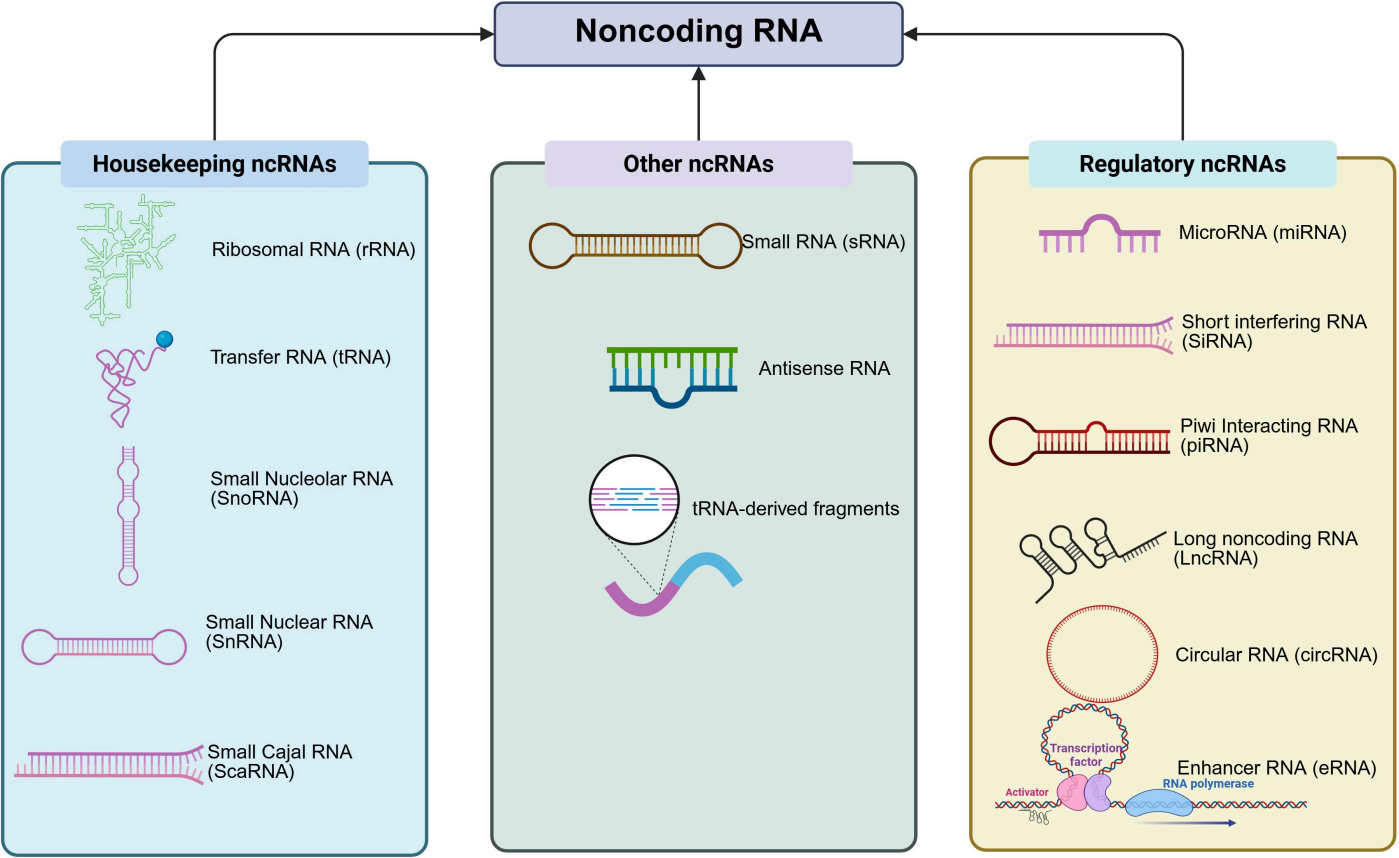
Classification of non-coding RNAs (ncRNAs). Non-coding RNAs are commonly categorized into housekeeping ncRNAs, regulatory ncRNAs, and other ncRNA classes based on their biological functions. Housekeeping ncRNAs include ribosomal RNA (rRNA), transfer RNA (tRNA), small nucleolar RNA (snoRNA), small nuclear RNA (snRNA), and small Cajal body–specific RNA (scaRNA), which are primarily involved in fundamental cellular processes such as protein synthesis and RNA processing. Regulatory ncRNAs include microRNA (miRNA), small interfering RNA (siRNA), PIWI-interacting RNA (piRNA), long non-coding RNA (lncRNA), circular RNA (circRNA), and enhancer RNA (eRNA), which participate in gene expression regulation at transcriptional and post-transcriptional levels. Additional ncRNA subclasses include small RNAs (sRNAs), antisense RNAs (asRNAs), and tRNA-derived fragments (tRFs), which contribute to diverse regulatory and cellular functions.

NcRNAs comprise a diverse class of functional RNA molecules that are not translated into proteins but have garnered significant biological attention because of their emerging roles as key regulators of multiple cellular functions in health and disease (25). These molecules are classified according to their structure, length, cellular localization, and function, encompassing lncRNAs, miRNAs, heterogeneous nuclear RNAs (hnRNAs), ribosomal RNAs (rRNAs), circular RNAs (circRNAs), small nuclear RNAs (snRNAs), small nucleolar RNAs (snoRNAs), transfer RNAs (tRNAs), and PIWI-interacting RNAs (piRNAs) (Figure 2) (25, 28, 29). Within the context of ADs, including aortic aneurysms, atherosclerosis, and AS, miRNAs and lncRNAs have emerged as particularly critical regulators of gene expression, inflammation, cell differentiation, and extracellular matrix remodeling, underscoring their potential as both diagnostic biomarkers and therapeutic targets (30, 31).

## LncRNA–miRNA crosstalk in aortic disease: between functional and pathological interplay

miRNAs are small non-coding RNAs (approximately 20–25 nucleotides in length) that regulate gene expression at the post-transcriptional level by binding to complementary sequences within messenger RNAs (mRNAs), leading to mRNA degradation or translational repression. Through this mechanism, miRNAs play crucial roles in cell proliferation, differentiation, apoptosis, immune responses, and the progression of cardiovascular diseases (11, 25, 30). On the other hand, lncRNAs are defined as non-coding transcripts exceeding 200 nucleotides that do not encode proteins but regulate gene expression through diverse mechanisms, including chromatin remodeling, transcriptional activation or repression, and post-transcriptional modulation (30, 31). A particularly significant mechanism of lncRNA function involves their ability to act as molecular sponges or inhibitors for miRNAs, a concept termed competitive endogenous RNAs (ceRNAs). By sequestering miRNAs, lncRNAs prevent their interaction with target mRNAs, thereby modulating gene expression networks (29–32). Because of these intrinsic regulatory properties, both miRNAs and lncRNAs have emerged as promising biomarkers and therapeutic targets in cardiovascular diseases.

The interplay between lncRNAs and miRNAs encompasses multiple diverse and complex regulatory mechanisms. Under pathological conditions, for example, lncRNAs such as MALAT1 (which sponges miR-204 and miR-195) (16, 33), TUG1 (which sponges miR-204-5p) (34), and NEAT1 (which sponges miR-214-3p) (35) promote osteogenic differentiation in human VICs, thereby contributing to CAVD progression. Conversely, other lncRNAs such as HOTAIR have been shown to ameliorate CAVD (36), highlighting the context-dependent nature of these interactions (29–32). Beyond their role as miRNA sponges, lncRNAs can also function as precursors for miRNA biogenesis, where an lncRNA transcript contains a stem-loop or hairpin structure that is processed into a mature miRNA (25, 28). Notable examples include H19 and MIR503HG, which serve as precursors for miR-675 and miR-503-3p, respectively, promoting osteoblastic transition and vascular smooth muscle cell (VSMC) proliferation *in vitro* (37, 38). Similarly, miR-15a, generated from the lncRNA AK058003, plays a key role in inflammation and calcification through the regulation of Human antigen R (HuR) (39).

**Figure 3:**
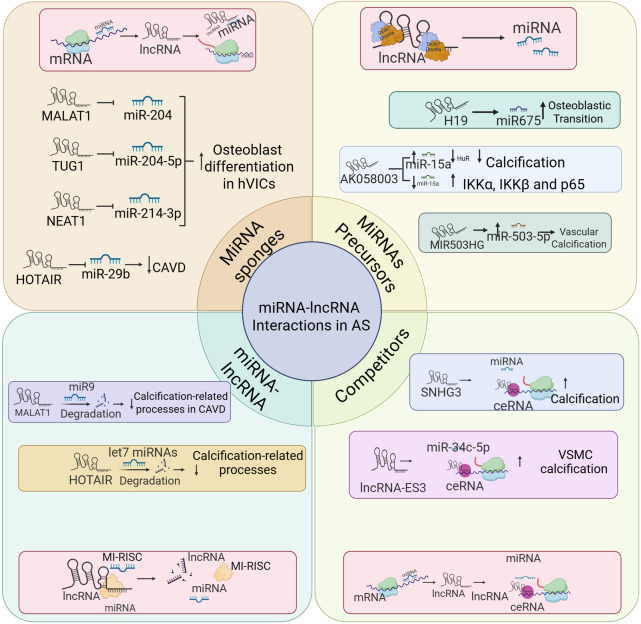
miRNA–lncRNA interactions in vascular calcification-related processes. A schematic illustration of the intricate interactions between lncRNAs and miRNAs involved in the pathogenesis of CAVD and vascular calcification. LncRNAs as sponges: lncRNAs such as MALAT1, TUG1, NEAT1, and HOTAIR interact with miRNAs (miR-204, miR-204-5p, miR214-3p, and miR-29b respectively) to promote osteoblast differentiation in hVICs and CAVD. LncRNAs as precursors: H19, AK058003, and MiR503HG and their respective products (miR675, miR-15a, miR-503-5p) are shown to directly affect cellular calcification processes. LncRNAs as competitors: SNHG3 and lncRNA-ES3 can act as ceRNAs to specific miRNAs (such as miR-34c-5p) to prevent them from binding to their target mRNAs, thereby modulating calcification. MiRNA-mediated lncRNA degradation: miRNAs can act directly on lncRNAs and promote their degradation through miRNA-Ago2 via the RISC pathway and can modulate pathogenic functions of lncRNAs such as MALAT1 and HOTAIR when they are degraded by miR9 and let7, respectively.

Another layer of regulatory complexity involves direct competition between lncRNAs and miRNAs for mRNA binding sites. In AS, for example, the lncRNA SNHG3 associates with Enhancer of Zeste Homolog 2 (EZH2) and physically interacts with the polycomb repressive complex 2 (PRC2) to upregulate BMP2 expression in aortic valve calcification (40). More broadly, competition arises when lncRNAs and mRNAs share common miRNA binding sites, allowing lncRNAs to act as ceRNAs that sequester miRNAs away from their mRNA targets (28). An illustrative example is lncRNA-ES3, which functions as a ceRNA for miR-34c-5p, thereby enhancing the expression of the target gene Bcl-2 modifying factor and promoting VSMC calcification (41). Conversely, miRNAs can act directly on lncRNAs to promote their degradation through the miRNA-Ago2-RISC pathway and in the context of CVDs and inflammation; such miRNA-mediated lncRNA degradation can modulate pathogenic lncRNA functions (29, 30). For example, degradation of MALAT1 by miR-9 and HOTAIR by let-7 miRNAs has been shown to help containing inflammation, fibrosis in diabetic cardiomyopathy, and calcification-related processes (Figure 3) (42–44).

These diverse and interconnected regulatory mechanisms between lncRNAs and miRNAs, including miRNA sponging, direct competition, miRNA-mediated degradation, and miRNA production from lncRNA precursors, establish complex feedback loops that significantly expand the complexity of gene regulatory networks. Given that their interplay represents a key factor in various disease states, targeting miRNA–lncRNA interactions may offer novel therapeutic strategies for complex pathologies such as cancer, neurodegenerative disorders, and cardiovascular conditions (28–30).

## LncRNAs in calcification pathways in aortic disease

LncRNAs have emerged as key regulators in the pathogenesis of AS, influencing molecular pathways involved in inflammation, oxidative stress, and calcification (28). They modulate AS through diverse mechanisms, including transcriptional regulation, chromatin remodeling, and post-transcriptional control, often interacting with transcription factors, miRNAs, and RNA-binding proteins (RBPs) to fine-tune gene expression (10–13, 28–30). For example, MALAT1 enhances endothelial dysfunction by regulating the Janus kinase/signal transducer and activator of transcription (JAK/STAT) pathway by counteracting the function of miR-590 (45), while H19 modulates periostin expression by inhibiting miR-let-7 (46). Other lncRNAs such as ANRIL (47) and XIST (48) exacerbate inflammation in valve tissues by modulating NF-κB signaling. In addition, the recently characterized lnc-COL6A1-6 is significantly upregulated in hVICs and may play a key role in autophagic activity during aortic valve calcification (49).

These observations position lncRNAs not only as potential biomarkers for AS progression but also as promising therapeutic targets. Elucidating their molecular roles may pave the way for novel interventions in AS management, with the potential to prevent disease progression and improve patient outcomes. As previously discussed, AS and related vascular calcification in humans are primarily driven by VICs, valvular endothelial cells (VECs), and smooth muscle cells (SMCs), with a lesser contribution from endothelial-to-mesenchymal transition (EndMT) processes (25, 31, 32). A growing body of evidence has identified specific lncRNAs that play distinct roles in regulating these pathways. In the following paragraphs, we detail key examples of lncRNAs involved in modulating AS and vascular calcification, incorporating relevant findings from human studies and animal models (Table 1).

**Table 1:** LncRNAs involved in promoting osteogenic differentiation and calcification in aortic valve disease.

LncRNA	Category	Mechanism of action	Reference
AFAP1-AS1	Procalcific	Triggers VIC calcification via miR-155 inhibition and SMAD5 upregulation	(50)
GAS5	Anticalcific	Overexpression of GAS5 inhibits vascular calcification via the GAS5/miR-26b-5p/PTEN axis, leading to AKT pathway suppression and reduced osteogenic gene expression in hVSMCs	(51)
H19	Procalcific	Drives VIC calcification in CAVD by inhibiting p53-mediated NOTCH1 activation, thereby derepressing osteogenic factors RUNX2 and BMP2	(52)
lnc-COL6A1-6	Procalcific	Upregulated expression in hVICs and samples from patients with CAVD. Inhibition *in vitro* alleviated calcification, ALPL activity, RUNX2, and osteopontin protein expression	(49)
MALAT1	Procalcific	Upregulated MALAT1 drives VIC calcification by sponging miR-204, leading to increased Smad4 expression and osteogenic fate commitment in hVICs. MALAT expression in hVSMCs promotes calcification by sponging miR-30c and RUNX2	(16, 53)
NEAT1	Procalcific	Upregulated in human calcified aortic valves; promotes VIC osteogenesis via the miR-214-3p/PTEN/PI3K-AKT pathway, leading to increased expression of osteogenic markers ALP and osteocalcin	(35)
OIP5-AS1	Anticalcific	Overexpression of OIP5-AS1 inhibits VIC osteogenic differentiation via the OIP5-AS1/miR-137/TWIST1 axis, leading to decreased ALP and osteocalcin levels	(54)
SNHG3	Procalcific	Upregulated in calcified aortic valve tissue from patients with CAVD. Binds the PRC2 complex and inhibits H3K27 trimethylation at the BMP2 promoter, thereby activating BMP2 expression and osteogenic signaling. *In vivo* silencing of SNHG3 reduces valve calcification in ApoE^⁻/⁻^ mice	(40)
TUG1	Procalcific	Highly expressed in calcified human valves and VICs. Sponges miR-204-5p to promote RUNX2 expression and calcification	(34)
MALAT1	EndMT	Upregulated MALAT1 promotes ox-LDL-induced EndMT in endothelial cells through activation of the Wnt/β-catenin pathway, resulting in elevated mesenchymal markers α-SMA and vimentin	(55)

VIC, valvular interstitial cell; EC, endothelial cell; CAVD, calcific aortic valve disease. This table summarizes experimentally validated lncRNAs implicated in aortic valve calcification and endothelial dysfunction. Procalcific lncRNAs promote VIC osteogenic differentiation through ceRNA networks (AFAP1-AS1/miR-155/SMAD5, MALAT1/miR-204/Smad4, TUG1/miR-204-5p/RUNX2), epigenetic regulation (SNHG3/PRC2/BMP2), transcriptional derepression (H19/p53/NOTCH1/RUNX2/BMP2), and PI3K-AKT pathway activation (NEAT1/miR-214-3p/PTEN). Anticalcific lncRNAs protect against calcification via GAS5/miR-26b-5p/PTEN/AKT and OIP5-AS1/miR-137/TWIST1 axes. EndMT-related lncRNAs drive endothelial-mesenchymal transition, exemplified by MALAT1-mediated Wnt/β-catenin activation in response to ox-LDL. References are indicated in parentheses.

## LncRNAs in inflammatory pathways

LncRNAs have been implicated in the regulation of endothelial function, cardiovascular physiology, and a spectrum of pathological conditions, including cardiac hypertrophy, myocardial infarction, and valvular disease progression (56–58). In particular, lncRNAs have emerged as pivotal regulators of inflammation, modulating inflammatory activity through diverse mechanisms that influence gene expression and involve interactions with key molecular players, thereby underscoring their importance as potential biomarkers and therapeutic targets (59, 60). Recent studies have revealed that several lncRNAs serve as essential components of classical immune pathways, including NF-κB signaling, the MAPK cascade, and the JAK/STAT pathway, positioning them as central regulators of inflammatory responses and tissue remodeling within the CV system (60, 61).

## LncRNAs in NF-κB signaling

**Figure 4:**
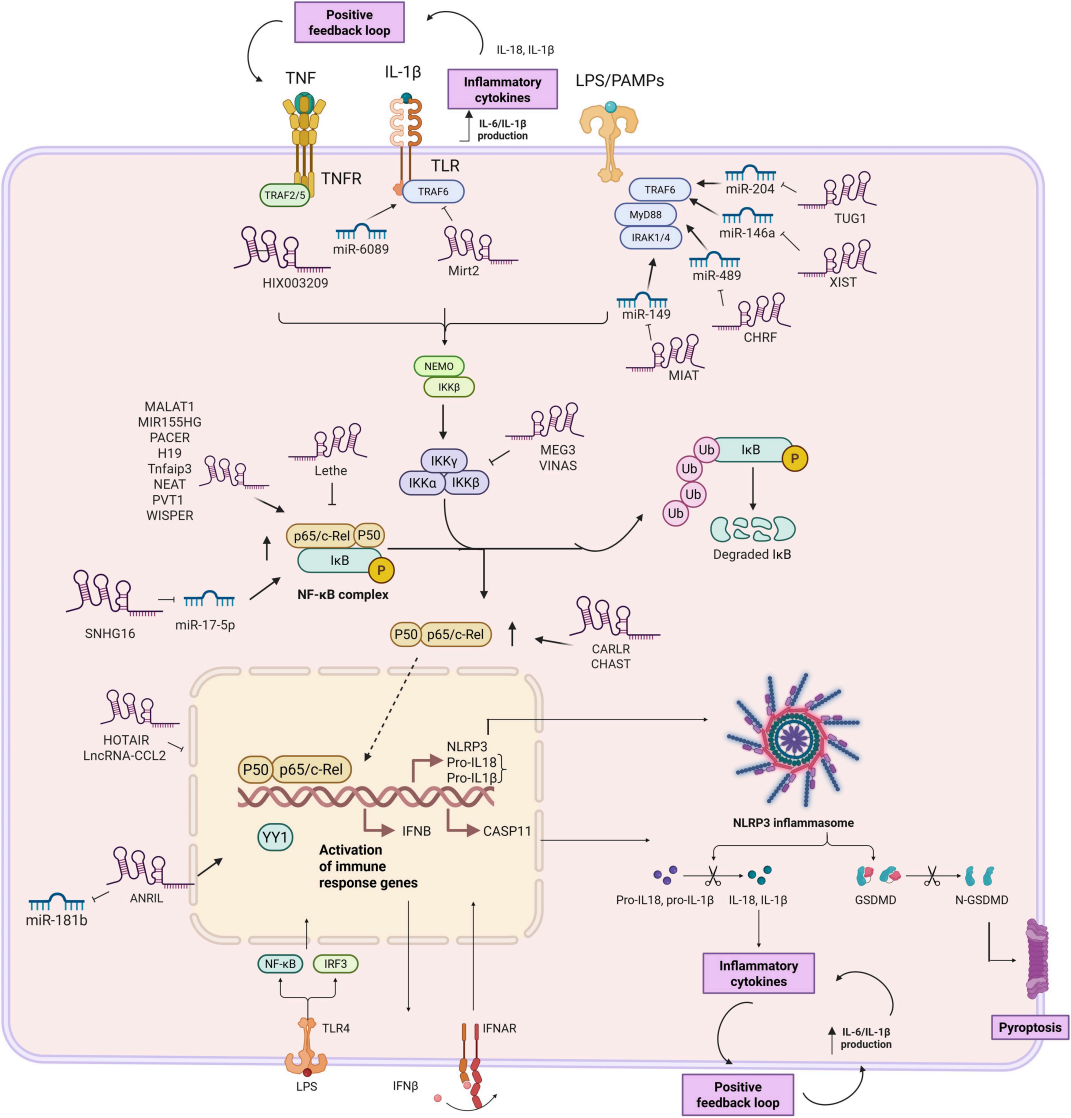
An integrated molecular pathway of lncRNAs regulating NFKb signaling. A schematic illustration of the integrated molecular pathways through lncRNAs modulating NF-κB signaling and inflammatory cascades in the context of AS. Proinflammatory cytokines (TNF-α, IL-1β, IL-18), toll-like receptors (TLRs), and other pattern recognition receptors (PRRs) activation engage downstream adaptors (TRAF2/5, TRAF6), leading to NF-κB complex activation and IκB degradation allowing nuclear translocation of the p50/p65/c-Rel complex. Key lncRNAs, including MALAT1, TUG1, H19, NEAT, and others, influence NF-κB activity and downstream effects, including the activation of immune response genes, NLRP3 inflammasome assembly, and caspase-1 activation, promoting the release of mature IL-18 and IL-1β, thereby establishing a positive feedback loop.

Emerging evidence in several CVDs highlights the role of lncRNAs in modulating the NF-κB signaling pathway, which is central to the inflammatory responses associated with AS (60, 61). One example is the LncRNA ANRIL, which is associated with T-cell activation, INFγ, and TNF-α increase in the context of uremic cardiomyopathy (62). The lncRNA CARLR enhances NF-κB pathway genes by promoting the transcription of proinflammatory genes in human monocytic cells under lipopolysaccharide (LPS) stimulation (63). Conversely, lncRNAs such as lincRNA-EPS function as a negative regulator of inflammation, suppressing immune-related genes in mouse macrophages (64). Figure 4 illustrates the dual regulatory capacity underlining the complex context-dependent roles of lncRNAs in fine-tuning inflammatory processes in AS. Similarly, Table 2 summarizes lncRNAs that have been reported to either activate or suppress NF-κB signaling in the context of AS or related cardiovascular conditions.

**Table 2:** LncRNAs involved in activating or suppressing NF-κB signaling in aortic stenosis and related cardiovascular conditions.

LncRNA	Mechanism of action	Interaction with NF-κB signaling	Reference
ANRIL	Associated with vascular diseases, including atherosclerosis, by influencing smooth muscle cell behavior and contributing to disease progression	Acts as a competing endogenous RNA for miR-181b mediating NF-κB signaling. Can interact with YY1 to enhance the expression of IL-6 and IL-8 in uremic cardiomyopathy	(47, 62, 65)
CARLR	May interact with chromatin-modifying complexes to regulate gene expression in cardiac cells, potentially influencing AVS-related pathways	Upregulation of IL-1β expression and *Ptgs2* genes activated *in vitro* by LPS in THP-1 cells	(63)
CHAST	Upregulated in hypertrophic heart tissue from patients with AVS, suggesting a role in cardiac hypertrophy associated with AVS	Potentially influences NF-κB signaling through the modulation of hypertrophic pathways	(66)
CHRF	Regulation of cardiac hypertrophy via miR-489 and Myd88	Acts as a sponge for miR-489, leading to the upregulation of Myd88, an adaptor protein that activates NF-κB signaling	(67)
H19	Elevated in atherosclerosis patients; augments oxidized LDL-induced lipid accumulation, ROS generation, and NF-κB activation in macrophages	Enhances NF-κB activation in response to oxidized LDL	(68, 69)
HIX003209	Interacts with the NF-κB signaling pathway in the context of rheumatoid arthritis (RA)	Functions as a ceRNA that sponges miR-6089, leading to the upregulation of TLR4/NF-κB	(70)
HOTAIR	Decreased in atherosclerosis patients; overexpression reduces proinflammatory cytokine expression by inhibiting NF-κB activity	Inhibits NF-κB activity by suppressing FXR1	(45, 71)
Lethe	A reduction of its expression determines an increased inflammation cascade in an *in vitro* model of hyperglycemia	Lethe overexpression reduced *NOX2* gene expression and p65- NF-κB translocation to the nucleus and limited ROS production	(72)
Tnfaip3	Required for transactivation of NF-κB-regulated inflammatory genes	Interacts with NF-κB subunit Hmgb1 to form a complex that modulates histone modifications, thereby influencing inflammatory genes in mouse macrophages	(73)
lncRNA-CCL2	Immune function regulated through lncRNA-CCL2 and downregulation of SIRT1 related to NF-κB	lncRNA-CCL2 and IL-1β, IL-6, and TNF-α increased after SIRT1 knockdown in LPS-stimulated macrophages	(74)
lincRNA-EPS	Localizes at the regulatory regions of immune response genes to repress transcription	Interacts with heterogeneous nuclear ribonucleoprotein L in mouse macrophages and regulates IL6 and INFγ	(64)
lncRNA lnc13	Regulates gene expression by binding to heterogeneous nuclear ribonucleoprotein D	Gene expression regulation by hnRNPD/p42/Hdac1 in mouse macrophages under LPS stimulation	(75)
MALAT1	Plays an important role in the injury caused by ischemia–reperfusion and diabetes in cardiomyocytes. Sponges miR-195 promoting tumorigenesis and immune escape of diffuse large B-cell lymphoma	MALAT1 knockdown alleviates myocardial inflammation induced by saturated fatty acids through the miR-26a/HMGB1/TLR4/NF-κB pathway. Important relationship between MALAT1/miR-195 axis in CAVD	(33, 76–79)
MEG3	Protects the intestinal barrier from cardiac arrest; MEG3 knockdown reduces stress-mediated myocardial apoptosis and cardiomyocyte hypertrophy	Interacts via the miR-34a-3p/SIRT1/NF-κB axis; regulates MMP-2 through a p53-dependent mechanism	(80, 81)
MIAT	Increased in patients with CAD; ablation of *Miat* attenuates pathological hypertrophy	Modulates the NF-κB pathway in cardiac hypertrophy	(82, 83)
MIR155HG	Promotes proliferation, migration, and antiapoptosis of MSCs	Regulates MSCs through the NF-κB pathway under oxidative stress	(84)
Mirt2	Reduction of proinflammatory cytokine production	Inhibits NF-κB and MAPK pathway activation in macrophages under LPS stimulation	(85)
NEAT1	Upregulated in atherosclerosis patients; contributes to proinflammatory responses by enhancing NF-κB activation; NEAT1 knockdown suppresses proliferation and induces apoptosis in HAECs treated with Ox-LDL	Upregulation mediated through p38 and NF-κB; regulates the miR-638/AKT/mTOR signaling pathway	(86–88)
PACER	Potential target for COX-2 modulation in inflammation	Interacts with NF-κB subunit p50 to enhance COX-2 expression	(89)
PVT1	Elevated in atherosclerosis patients; inhibition reduces atherosclerotic plaques by suppressing the MAPK/NF-κB pathway; cardiac hypertrophy is positively regulated by PVT1	Modulates NF-κB signaling through the MAPK pathway; PVT1 knockdown suppresses Gasdermin D-mediated pyroptosis in myocardial ischemia	(90–92)
SNHG16	Amplifies macrophage proliferation and proinflammatory responses in atherosclerosis; knockdown reduces myocardial ischemia/reperfusion injury in rats	Sponges miR-17-5p, leading to increased NF-κB activity; silencing suppresses GSDMD-mediated pyroptosis	(93)
TUG1	Promotes VSMC proliferation in atherosclerosis; promotes cardiac fibroblast activation and fibrosis by upregulating collagen synthesis; overexpression exacerbates myocardial injury and AS	Acts as a ceRNA for miR-21, modulating the PTEN pathway; targets miR-29c in chronic hypoxia	(34, 94–96)
VINAS	Regulates vascular inflammation, contributing to the development of atherosclerosis	VINAS downregulation decreases the expression of VCAM-1, E-selectin, ICAM-1, TNF-α, and IL-1β in ECs, VECs, and bone marrow–derived macrophages	(97)
WISPER	Correlated with cardiac fibrosis in murine models and patients with AVS, suggesting a role in fibrotic processes	Fibrosis modulation may indirectly affect NF-κB signaling: mice treated with antisense oligonucleotides to Wisper after infarction reduced collagens and cardiac stress markers	(98)
XIST	Main role in regulating myocardial infarction, cardiomyocyte hypertrophy *in vitro* using cardiomyocytes, and inflammation	Xist promoted expression of the calcium binding protein S100B through miR-330-3p, preventing cardiac hypertrophy. Targets miR-130a-3p promoting myocardial infarction	(99–101)

A summary of experimental evidence with lncRNAs as key players in the NF-κB signaling pathway. This table provides a mechanism of NF-κB modulation (direct or indirect), downstream target genes/effectors, and functional consequences on inflammation, calcification, and/or disease progression.

## LncRNAs in MAPK signaling

LncRNAs have emerged as key regulators of mitogen-activated protein kinase (MAPK) signaling, a critical pathway governing cellular processes including proliferation, differentiation, apoptosis, and stress responses (102–104). Their interactions with the MAPK cascade have garnered significant attention because of their contribution to pathological conditions such as CVDs, immune disorders, and AS (104–106). In the context of AS, lncRNAs have been implicated in the osteogenic differentiation of VICs, a key process driving valve calcification (102). For example, the TUG1 promotes osteoblastic differentiation in VICs by sponging miR-204-5p, leading to the upregulation of Runx2, a master transcription factor in osteogenesis (34). Similarly, the human orthologue of mouse VINAS, DEPDC4, has been shown to promote NF-κB/MAPK-driven cytokine and adhesion molecule expression; knockdown of this lncRNA in endothelial and SMCs reduces p38 MAPK phosphorylation and decreases atherosclerotic lesion formation by approximately 55%, processes closely linked to osteogenic fate and calcification (97). The well-characterized MALAT1, which exhibits sustained upregulation and leads to a p38-sustained phosphorylation in a diabetes mellitus contexts, is also highly expressed in patients with AS, suggesting a pathological role linked to MAPK signaling (107). Direct modulators of the MAPK pathway include lncRNA-MAP3K4, which is upregulated in endothelial cells during inflammation and modulates MAPK signaling to affect the expression of proinflammatory cytokines such as IL-1β and TNF-α (107, 108). Other lncRNAs such as SNHG3 function as negative regulators of inflammation, oxidative stress, and apoptosis by sponging miR-330-5p and regulating the ERK/p38 axis, as demonstrated in a myocardial ischemia–reperfusion injury model (109). These findings highlight the complex and context-dependent functions of lncRNAs in CV pathologies and underscore their potential as therapeutic targets in conditions such as AS.

**Figure 5:**
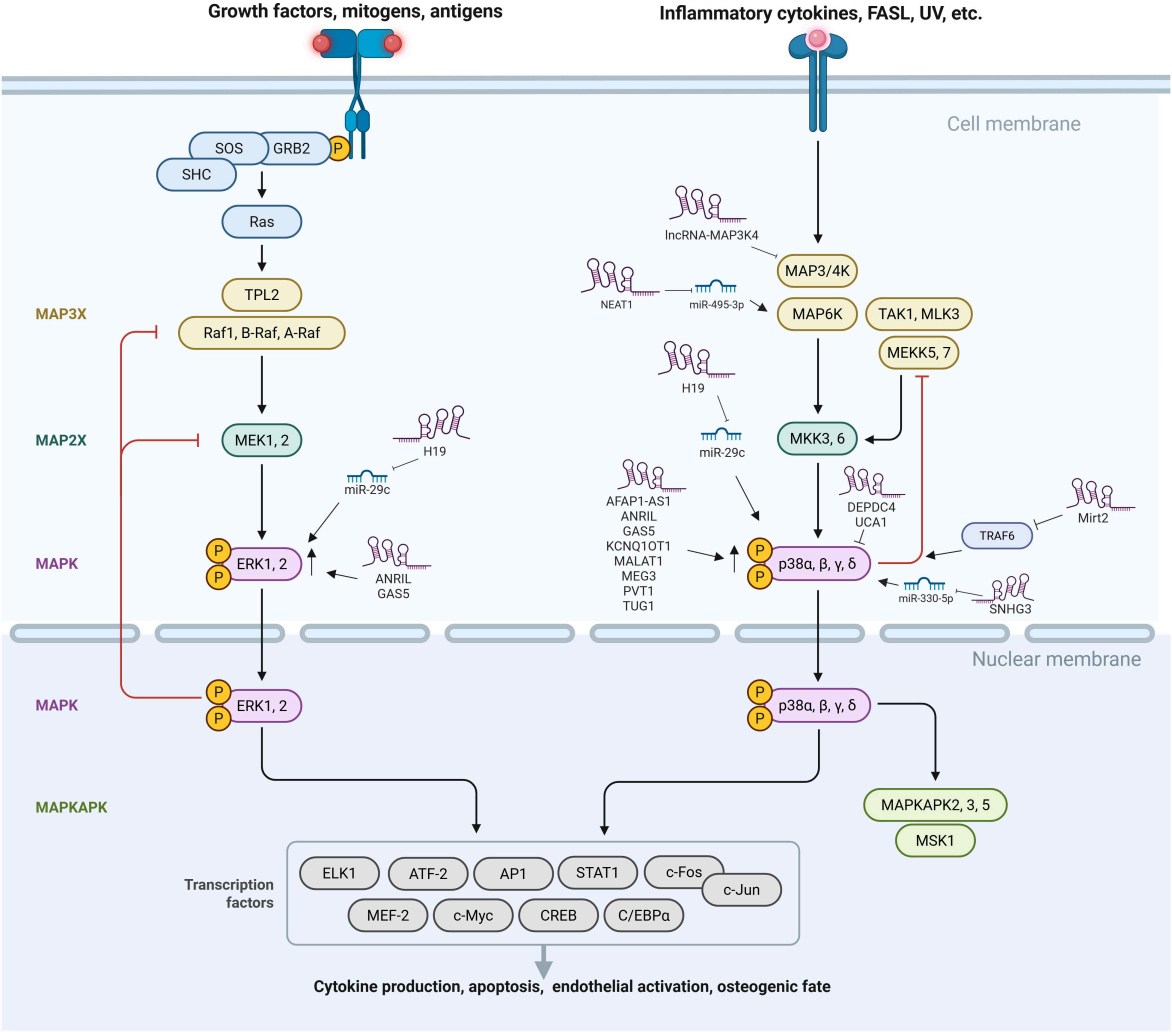
Integrated molecular pathways of lncRNAs regulating MAPK signaling. A schematic diagram representing the major components of MAPK signaling cascades and their modulation by lncRNAs and miRNAs. Key lncRNAs (e.g., H19, MALAT1, NEAT1, SNHG3, TUG1, GAS5, and ANRIL) and miRNAs (e.g., miR-29c, miR-330-5p, and miR-495-3p) are shown interacting with specific pathway components, including upstream adaptors (SHC, GRB2, SOS), small GTPases (Ras), and kinases (Raf, MEK, TAK1, TRAF6). These interactions ultimately regulate the activity of transcription factors (ELK1, c-Jun, c-Fos, c-Myc, CREB, ATF-2, AP1, STAT1, MEF-2, C/EBPα) that control downstream cellular processes such as cytokine production, apoptosis, endothelial activation, and osteogenic fate determination.

These multiple regulatory effects are mediated by multiple mechanisms, including cis-regulation of the MAPK cascade, sponging of miRNAs that target MAPK effectors, and epigenetic modulation (Figure 5) (102–125). In the following paragraphs, we present a summary of lncRNAs that have been reported to either activate or suppress MAPK signaling in the context of AS and related cardiovascular conditions (Table 3).

**Table 3:** LncRNAs involved in regulating MAPK signaling and inflammation in AS.

LncRNA	Mechanism of action	Interaction with MAPK signaling	Reference
AFAP1-AS1	Promotes p38 (via RhoC/ROCK1) and regulates cytokine production and endothelial activation; promotes tumorigenesis and epithelial–mesenchymal transition of osteosarcoma cell lines and BALB/c mice	Acts as a molecular sponge (e.g., for miR-7-5p) to repress upstream RTKs like EGFR; acts through the RhoC/ROCK1/p38 MAPK/Twist1 signaling pathway in the epithelial–mesenchymal transition of osteosarcoma	(111, 112)
ANRIL	Robustly engages MAPK signaling, suggesting it could similarly influence inflammatory MAPKs in cardiovascular conditions	Boosts p-ERK, p-p38, and p-JNK levels in carcinoma models; regulates tumorgenesis through FGFR1 and sponging miR-125a-3p	(113–115)
GAS5	Modulates MAPK signaling during myocardial ischemia–reperfusion injury, promoting inflammation and cardiomyocyte apoptosis	Overexpression activates p38 and ERK MAPK signaling in mice via binding to miR-532-5p	(116)
H19	Modulates apoptosis; melatonin's cardioprotective effects involve the H19/miR-29c/MAPK axis	Acts as a ceRNA for miR-29c, derepressing ERK1/2 and the p38 MAPK pathway in murine models	(117)
KCNQ1OT1	Drives injury via the p38 MAPK/NF-κB pathway in myocardial ischemia/reperfusion injury	Silencing downregulates the phosphorylation of p38 MAPK (p-p38)	(118)
lncRNA-MAP3K4	Shows coordinated expression with MAP3K4 following vascular inflammatory induction in ECs and VECs *in vitro*	Regulates vascular inflammation through the p38 MAPK signaling pathway and TNF-α, IL-1β, and COX2 expression	(119)
MALAT1	Activates p38 MAPK without affecting ERK1/2 or JNK; enhances IL-8/TNF-α production and EC migration	Sustained p38 phosphorylation by upregulated MALAT1 in diabetes mellitus	(107, 108)
MEG3	Plays a role in inflammation during viral and cardiac injury by modulating the p38 MAPK pathway via miR-21	Regulates viral replication via the lncRNA MEG3/miRNA-21/p38 MAPK axis in an acute viral myocarditis model	(120)
Mirt2	Inhibits the MAPK pathway activation and downregulates inflammation in LPS-activated macrophages	Prevents activation of JNK, ERK1/2, and p38 MAPKs by binding the E3 ligase TRAF6	(85)
NEAT1	Overexpressed in mouse heart after ischemia–reperfusion, promoting inflammatory cytokine release	Sponges miR-495-3p, leading to MAPK6 activity and production of TNF-α, IL-1β, and IL-18	(121)
PVT1	Upregulated in inflammatory and septic cardiac injury models; knockdown inhibits p38 MAPK activation in THP-1-derived macrophages	Promotes TNF-α, IL-1β, and IL-6 release via p38 MAPK activation	(122, 123)
SNHG3	Modulates the ERK/p38 MAPK pathway to reduce inflammation	Sponges miR-330-5p regulating ERK/p38, reducing apoptosis and oxidative stress in a myocardial ischemia–reperfusion injury model	(109)
TUG1	May activate the JNK and p38 MAPK pathways to drive inflammation and AS in the heart	Activates the JNK and p38 MAPK pathways, promoting inflammation and cell death in a cerebral ischemia and reperfusion injury model	(34, 124)
UCA1	Upregulated in the serum of patients with AS hECs stimulated with ox-LDL	UCA1 sponges miR-873-5p; silencing UCA1 protects against ox-LDL-induced EC injuries through the miR-873-5p/MAPK8 axis in AS	(125)
VINAS	Knockdown in mice decreases proinflammatory and adhesion molecules	Knockdown reduces the phosphorylation of p38 MAPK in endothelial cells and smooth muscle cells	(97)

These studies highlight the important influence of lncRNAs on inflammation-related MAPK signaling cascades and their potential role in AS, opening new windows for targeted research aimed at modulating MAPK-driven pathways in AS and related cardiovascular diseases.

## LncRNAs in JAK/STAT signaling

**Figure 6:**
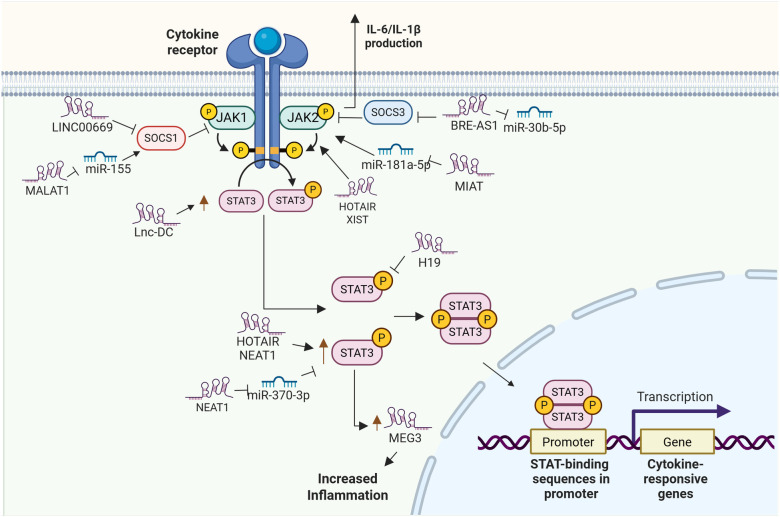
LncRNAs in JAK/STAT pathway signaling and their influence on the immune response. A schematic representation of the JAK/STAT signaling pathway activated by cytokine receptor engagement, highlighting regulatory inputs from lncRNAs and miRNAs. Following cytokine stimulation, STAT proteins (primarily STAT3 and STAT4) dimerize and translocate into the nucleus to bind STAT-responsive promoter elements, driving gene transcription and subsequent IL-6 and IL-1β production. Multiple lncRNAs modulate this pathway at different levels: LINC00669, MALAT1, and Lnc-DC regulate upstream components; while HOTAIR, XIST, MEG3, MIAT, and H19 influence STAT3 activity. LncRNAs such as LINC00669 and BRE-AS1 can act negatively by inhibiting SOCS1 and SOCS3, respectively. This intricate regulatory network may control the magnitude and duration of inflammatory gene expression in AS and/or cardiovascular diseases.

In the context of inflammation and CVDs including AS, diverse lncRNAs have been implicated in the modulation of the Janus kinase/signal transducer and activator of transcription (JAK/STAT) pathway (126, 127). Despite the situation that detailed research of the molecular mechanisms have been studied in different biological models (128) other very well-known actors like H19 can influence inflammatory pathways in other organs like the brain (129). Similar regulatory pathways might tame the events involved the CV system, where they may govern inflammation, AS progression, and related pathologies (126–130). LncRNAs can modulate JAK/STAT signaling through multiple mechanisms. Some lncRNAs upregulate cytokine receptor expression or directly enhance the phosphorylation of STAT proteins, thereby potentiating pathway activation. Others act as molecular sponges for miRNAs that target JAK/STAT components, thereby suppressing pathway activity. In addition, certain lncRNAs can function as molecular scaffolds, bringing together multiple proteins to either promote or terminate downstream signal propagation (Figure 6). For example, lncRNA BRE-AS1 has been shown to modulate inflammation in human THP-1 macrophages, where its silencing promotes enhanced JAK2/STAT3 activation and increased IL-6/IL-1β production via a miR-30b-5p-dependent mechanism (131). Similarly, upregulation of the lncRNA MIAT, through its inhibitory effect on miR-181a-5p, leads to higher JAK2/STAT3 signaling, inflammation, and apoptosis in oxygen–glucose-deprived cardiomyocytes (132). In the context of CV injury, a transcriptomic analysis of human samples has revealed that XIST upregulation correlates with JAK2 levels, suggesting that XIST promotes postischemic inflammation via the JAK2 axis (133). Another illustrative example studied in a different biological model is LINC00669, which promotes persistent activation of JAK/STAT signaling by competitively binding to the suppressor of cytokine signaling pathway 1 (SOCS1), thereby activating STAT1 and initiating the transcription of genes related to proliferation and invasion in nasopharyngeal cancer (134). Although many of these lncRNAs, including well-characterized examples such as MALAT1 and AGAP1-AS1, have been studied in both human and animal models, their precise roles in inflammation-related cardiovascular conditions and AS are still being elucidated (135, 136). In the following section, we compile and highlight lncRNAs that influence the JAK/STAT pathway within the context of inflammation, AS, and related cardiovascular conditions (Table 4).

**Table 4:** LncRNAs involved in regulating JAK/STAT signaling and inflammation in CVD.

LncRNA	Mechanism of action	Interaction with JAK/STAT signaling	Reference
BRE-AS1	Knockdown of BRE-AS1 increases IL-6/IL-1β production in human THP-1 macrophages under LPS stimulation	BRE-AS1 sponges miR-30b-5p and binds to SOCS3 to regulate JAK2/STAT3 activation	(131)
H19	Reduces IL-6 release and STAT3 activity, ICAM1 and VCAM1 expression. Downregulation decreases the expression of TNF-α and IL-1β in ECs	Loss of function of H19 upregulates p16 and p21, reduces proliferation, and increases senescence *in vitro*. STAT3 is repressed after H19 overexpression	(137)
HOTAIR	HOTAIR Promotes Myocardial Fibrosis: Effects on Cardiac Fibroblasts In Vitro and in an Angiotensin II Mouse Model	HOTAIR and Wnt5a upregulated in the Ang II model. Silencing HOTAIR represses Wnt5a via ERK/JNK, thereby inhibiting fibrosis	(138)
HOTAIRM1	Upregulated in HUVECs after ox-LDL stimulation *in vitro*	HOTAITM1 binds to the HOXA4 promoter in a positive feedback loop; regulatory mechanism under ox-LDL stimulation through the HOXA4/HOTAIRM1/HSPA5 axis	(139)
LINC00669	Related to immune response and proliferation in human nasopharyngeal carcinoma	Competitively binds to SOCS1, promoting STAT1 activity and JAK/STAT signaling activation, enhancing nasopharyngeal cancer	(134)
Lnc-DC	Crucial role in the differentiation and function of dendritic cells (DCs); DC-mediated T-cell activation through STAT3; upregulated in patients with CAD	Promotes STAT3 phosphorylation during DC differentiation; strong correlation between the expressions of lnc-DC, SCOCS1, and STAT3	(140, 141)
MALAT1	Reduces cytokine release and relieves inflammation in human coronary artery endothelial cells (HCAECs)	Suppresses inflammatory response and cell apoptosis by sponging miR-155 and increasing SOCS1 under an aox-LDL-induced apoptosis assay of HCAECs	(142)
MEG3	Upregulation mediated by STAT3 modulates inflammatory response in cardiac hypertrophy	STAT3 upregulates MEG3 expression through the miR-361-5p/HDAC9 axis	(143)
MIAT	Induces inflammation and apoptosis in oxygen–glucose deprivation (ischemia model) in rat cardiomyocytes	Sponges miR-181a-5p to enhance JAK2 expression and inflammation	(132)
NEAT1	Increased in AS clinical samples involved in exacerbating atherosclerosis development and inflammation	Modulates STAT3 activity by sponging miR-370-3p in HUVECs *in vitro* and promoting the expression of FMO3	(144)
TERC	Increased expression with osteogenic or inflammatory stimuli. Non-canonical TERT activity along with lncRNA TERC initiates osteogenesis in CAVD in *Tert^−/−^*mice	Non-canonical osteogenic functions along with STAT5 and binds to the RUNX2 gen promoter	(22)
XIST	Correlated with increased inflammation in acute myocardial infarction	Upregulation correlates with high JAK2/STAT expression; sponges miR-146a and activates JAK/STAT signaling	(133, 145)

A summary of known lncRNAs that modulate the JAK/STAT signaling pathway in the context of cardiovascular inflammation and related pathologies. Mechanisms include miRNA sponging, direct binding to SOCS family proteins, and regulation of STAT phosphorylation.

## Clinical significance, diagnostic potential, and current challenges

**Figure 7:**
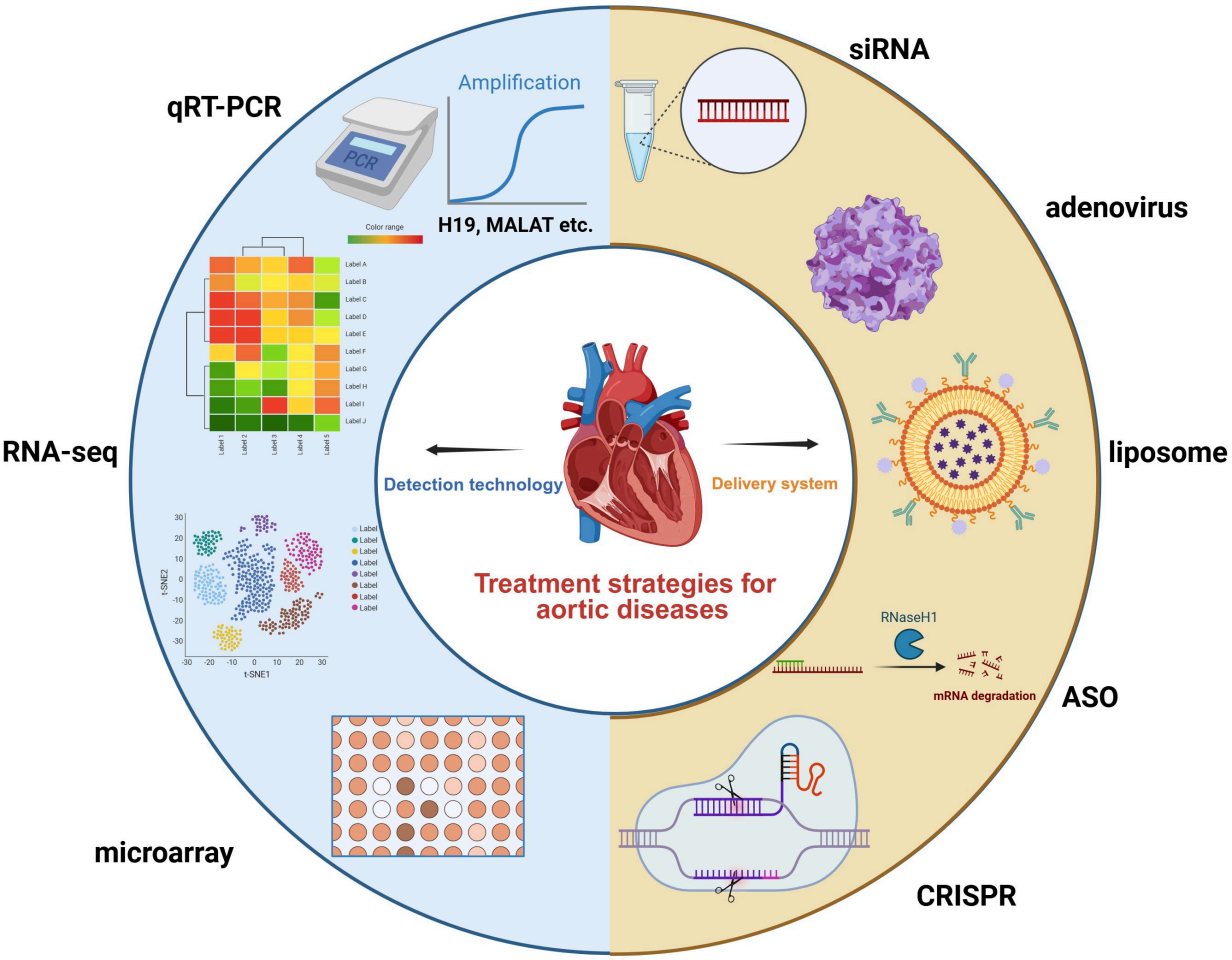
A schematic overview of LncRNA-based therapeutic strategies and detection platforms for ADs. This figure illustrates the integrated landscape of lncRNA-based approaches in aortic diseases, including AS. Key lncRNAs implicated in pathogenesis (e.g., H19 and MALAT1) are identified using detection platforms such as quantitative real-time PCR (qRT-PCR), RNA sequencing (RNA-Seq), and microarrays, enabling biomarker discovery and transcriptomic profiling (left panel). Therapeutic strategies targeting these lncRNAs (right panel) comprise three main modalities: antisense oligonucleotides (ASOs), which recruit RNase H1 for target degradation; small interfering RNAs (siRNAs), which mediate gene silencing via the RNA-induced silencing complex (RISC); and CRISPR-based genome editing for precise modulation of lncRNA expression. Liposomal nanoparticles and adenoviral vectors, underscoring their critical role in enabling effective lncRNA-targeted therapy, facilitate delivery. This schematic reflects the current technological framework and translational potential for lncRNA-based diagnostics in ADs.

Given the increasing global burden of AS, early identification and timely intervention are critical to preventing irreversible cardiac damage and improving patient outcomes (146–148, 149). In this context, lncRNAs have emerged as promising diagnostic and prognostic biomarkers because of their remarkable stability, tissue-specific expression patterns, and condition-dependent regulation in biofluids such as blood, plasma, and urine as seen in Figure 7 (146, 151). Within AS and related cardiovascular diseases, lncRNAs regulate key pathological processes, including inflammation, calcification, ECM remodeling, and smooth muscle cell dysfunction, which are central to disease progression (28–32, 150, 151).

Preclinical studies have demonstrated that modulating lncRNA expression can ameliorate disease phenotypes; for example, lncRNA FGD5-AS1, classically related to cancer progression, has shown to play a key role in cardiomyocyte apoptosis and inflammation in an *in vivo* model of acute myocardial infarction, whereas its expression sponges miR-223-3p and ameliorates mRNA expressions of IL-6, TNF-α, and IL-8 (152). Interestingly, in ApoE^−/−^ mouse, CAVD induced via high cholesterol diet was reduced by enhancing lncRNA FGD5-AS1 and/or repressing miR-497-5p (153), while other lncRNAs such as TUG1 (21, 34) and SNHG3 (40) have been suppressed, highlighting the therapeutic potential of lncRNA-based strategies (26–32).

A transcriptomic analysis of calcified aortic valves and other human tissue types has revealed consistent dysregulated levels of lncRNAs (103, 154, 155). A perfect example is MALAT1, whose expression is significantly increased in calcified leaflet tissues, and it exhibits a gradually increasing trend during the osteogenic induction of VICs and the expression levels of osteogenic-specific genes such as ALP and osteocalcin (16, 53). On the other hand, transcriptomic profiling studies (bulk RNA sequencing, microarrays) have also identified panels of lncRNAs that are differentially expressed between aortic valve disease and normal valves, providing potential candidates for biomarker detection (151, 154–156). However, few of them have been validated as reliable circulating biomarkers in human cohorts, highlighting the critical role of these molecules in AS pathogenesis and underscoring their potential as therapeutic targets (102, 103).

Although lncRNAs show promise as biomarkers, several challenges must be addressed before their widespread clinical application. One key challenge is the standardization of detection techniques. Current methodologies such as quantitative real-time polymerase chain reaction (qRT-PCR), RNA sequencing, and microarrays exhibit variability in sensitivity and specificity across different studies and clinical settings. In addition, the reproducibility of lncRNA biomarkers needs further validation in large-scale, multicenter studies. Another challenge is the need to establish robust reference databases for normal vs. pathological lncRNA expression levels, allowing for better interpretation of patient data.

## RNA therapeutics in cardiovascular disease

LncRNAs play a pivotal role in the pathogenesis and progression of AS. Currently, multiple lncRNA-targeted therapeutic approaches are under development. Depending on the desired molecular outcome, two primary strategies for lncRNA-based therapeutic interventions are being explored. The first strategy involves lncRNA antagonism, which can be achieved through short hairpin RNA (shRNA), siRNA, conformational small RNAs (aptamers), antisense oligonucleotides (ASOs), gapmeRs, or CRISPR-Cas9 genome editing (11).

## Antisense oligonucleotides

ASOs are short, single-stranded DNA or RNA molecules, typically 8–50 nucleotides in length, designed to bind specifically to target RNAs via sequence complementarity. ASOs exhibit high target specificity through base pairing, enabling discrimination of even single-base mismatches. Mechanistically, ASO-RNA hybrids are recognized by RNase H1, which cleaves the RNA transcript, thereby degrading the target lncRNA. Unlike siRNAs, ASOs do not require the RNA-induced silencing complex (RISC), allowing them to target both mature and precursor RNA transcripts. Furthermore, chemical modifications can enhance ASO stability, binding affinity, and cellular uptake, improving their therapeutic potential (157). For instance, specific engineered ASOs such as oligonucleotide long non-coding RNA MALAT1 (ASO-MALAT1) can suppress angiotensin II (AngII)-induced reactive oxygen species (ROS) production as well as proliferation and migration of VSMCs by inducing the expression of glutathione peroxidase 4 (GPX4) in an Ang II-induced abdominal aortic aneurysm murine model (158), thereby providing insights and/or foundations for lncRNA-based targeted therapies in AS and other CV conditions.

## Small interfering RNAs

Small interfering RNAs (siRNAs) are short, double-stranded RNA fragments that mediate target silencing via the RNA interference (RNAi) pathway. Mechanistically, the siRNA duplex is unwound within cells, and one strand is incorporated into the RNA-induced silencing complex (RISC), which then binds to and cleaves target RNA molecules in a sequence-specific manner, leading to post-transcriptional gene silencing (25–27). Studies have shown that siRNA exhibits remarkable silencing efficiency, with effects lasting for weeks even at picomolar (pM) concentrations. In mammalian cells, the cleaved RNA products are rapidly and completely degraded, making siRNA a powerful tool for both functional gene studies and the development of targeted therapies. Currently, siRNA technology has been widely applied in lncRNA functional research and the exploration of novel treatments for cardiovascular and other diseases (27–29). SiRNAs have been used to inhibit fibro-calcific differentiation of hVICs *in vitro* by targeting specific lnRNAs such as lncTSI, alleviating aortic valve calcification by negatively regulating the TGF-β/Smad3 pathway (159). Another example shows VICs under cyclic stretch conditions and treated with siRNAs against HOTAIR, resulting in an increased expression of calcification genes, cytokine response, leukocyte migration, and ossification (36). By harnessing the sequence-specific siRNA technology to achieve sustained transcriptional gene silencing, this alternative serves as a pivotal tool for modulating siRNAs and their potential downstream effects for formulating potential therapeutic strategies for complex fibro-calcific and cardiovascular pathologies.

## CRISPR-based gene editing

The CRISPR-Cas9 system has emerged as a transformative tool for precise genome editing (160). CRISPR-based approaches can be employed to knock out or modulate lncRNA expression in different biological systems (161). For instance, targeted disruption of procalcific lncRNAs could prevent or slow valve calcification, thereby reducing disease progression (161, 162). This approach offers the advantage of permanent genetic modification, which may be particularly valuable for chronic conditions such as AS, although it also raises important considerations regarding off-target effects and long-term safety.

## Delivery of RNA therapy

Delivery vehicles for lncRNAs are broadly categorized into viral and non-viral systems (163). Lentiviral vectors, renowned for their high transfection efficiency and sustained gene expression, are widely employed in experimental settings to modulate lncRNA levels, either through overexpression of specific lncRNAs or delivery of siRNAs for targeted knockdown (162). Adenoviral vectors, characterized by high transduction efficiency and transient expression, serve as another viable option (163, 164). Among non-viral systems, lipid-based nanoparticles (e.g., liposomes and nanoliposomes) have gained prominence in preclinical research. Their encapsulation capacity enhances drug stability, improves hepatic targeting by evading renal filtration, and reduces toxicity and immunogenicity (165). In addition, extracellular vesicles (EVs) like exosomes show considerable promise in modulating pathogenic lncRNA expression (166, 167).

Although lncRNAs have emerged as pivotal regulators of gene expression with substantial therapeutic potential, their clinical translation faces several hurdles. A major challenge lies in achieving tissue-specific delivery, as lncRNAs often engage in complex interactions with multiple targets, making the development of selective and efficient delivery systems a persistent obstacle (168–171). Furthermore, a comprehensive understanding of lncRNA functions in CVDs remains incomplete (168). Although significant progress has been made in identifying lncRNA biomarkers, elucidating their intricate regulatory networks, including interactions with protein-coding genes and non-coding elements, is essential to decipher their functional roles in disease progression. Technologically, optimizing delivery systems through chemical modifications, engineered nanoparticles, and other strategies is critical to enhance lncRNA stability, delivery efficiency, and biosafety. Looking ahead, lncRNAs hold transformative potential for diagnosing and treating AS and other CVDs, but their clinical application requires overcoming technical and biological challenges. Key priorities include large-scale clinical validation, refinement of delivery platforms, and development of combinatorial therapies targeting shared pathways. As research advances, lncRNA-based therapeutics may open new avenues for precision medicine, provided rigorous preclinical evaluation and robust safety profiling underpin their translation.

## Current challenges and limitations

Despite the promise of ncRNAs, particularly lncRNAs and miRNAs, as biomarkers and potential therapeutic targets in AS, and related cardiovascular diseases, several critical technical, biological, and translational challenges must be addressed before their widespread clinical application can be realized (169, 172). From a diagnostic perspective, a primary obstacle is the standardization of detection techniques. Current methodologies, including quantitative real-time PCR (qRT-PCR), RNA sequencing, and microarrays, exhibit substantial variability in sensitivity, specificity, normalization strategies, sample processing, and analytical pipelines across different studies and clinical settings. In addition, the reproducibility of candidate ncRNA biomarkers requires rigorous validation in large-scale, multicenter cohorts to confirm their diagnostic, prognostic, and risk-stratification value (29, 169, 172). Another important requirement is the establishment of robust reference databases that define physiological vs. pathological ncRNA expression patterns across different ages, sexes, comorbidities, disease stages, and tissue or biofluid sources, thereby enabling more accurate interpretation of patient-derived data (29–32). Beyond these technical issues, fundamental biological questions remain regarding ncRNA-mediated regulation of gene expression in physiological and pathological cardiovascular states. For miRNAs, numerous targets involved in inflammation, fibrosis, osteogenic differentiation, and extracellular matrix remodeling have been described; however, target identification and validation remain complicated by the context-dependent nature of miRNA regulation. A single miRNA may regulate multiple mRNAs, whereas individual mRNAs may be cooperatively regulated by several miRNAs (155–157). These combinatorial interactions may differ across cell types, disease stages, and species, making it difficult to assign causality to specific miRNA–mRNA pairs in complex diseases such as AS (172). Similar challenges apply to lncRNAs, whose functions are often highly dependent on cellular context, subcellular localization, transcript abundance, binding-partner availability, and disease microenvironment. A given lncRNA may exert distinct or even opposing effects in valvular interstitial cells, valvular endothelial cells, macrophages, vascular smooth muscle cells, or osteoblast-like cells, and its function may be modified by mechanical strain, extracellular matrix stiffness, oxidative stress, inflammatory cytokines, and calcific burden (155, 169).

Several lncRNA-specific biological features further limit direct therapeutic translation. Many lncRNAs display poor evolutionary conservation compared with protein-coding genes, and orthologous transcripts may be absent, weakly conserved, or functionally divergent across species. This complicates extrapolation from murine and other animal models to human valve disease. This limitation is particularly relevant in AS, where the human aortic valve is exposed to decades of mechanical stress, lipid deposition, inflammation, extracellular matrix remodeling, and progressive mineralization, features that are only partially reproduced in commonly used preclinical models. Moreover, much of the current evidence in the AS field relies on *in vitro* osteogenic differentiation of valve interstitial cells, bulk tissue analyses, and a limited number of animal studies, whereas validation in independent human valve cohorts, spatially resolved tissue sections, single-cell datasets, and *ex vivo* valve models remains comparatively limited (19, 24, 27).

Functional interpretation is also complicated by the frequent use of non-physiological overexpression or knockdown systems. Although siRNA, shRNA, ASO, gapmeR, viral overexpression, and CRISPR-based approaches are indispensable for target discovery, they may not accurately reproduce endogenous lncRNA abundance, isoform usage, subcellular localization, or physiological binding-partner stoichiometry (168, 172). Overexpression may generate artificial RNA–RNA or RNA–protein interactions that do not occur *in vivo*, whereas knockdown approaches may cause incomplete depletion, off-target effects, compensatory responses, or activation of innate immune pathways. These concerns are particularly important for proposed competing endogenous RNA mechanisms, because lncRNA–miRNA–mRNA interactions depend strongly on relative transcript abundance and stoichiometric balance. Therefore, future mechanistic studies should incorporate multiple independent perturbation strategies, dose–response analyses, rescue experiments, subcellular localization assays, and validation at endogenous expression levels. From a therapeutic perspective, achieving tissue- and cell-type-specific delivery remains a major unresolved challenge. LncRNAs frequently interact with multiple RNA, DNA, and protein partners, making selective modulation difficult. Technological advances, including chemical modifications, engineered nanoparticles, extracellular vesicles, and other delivery systems, may improve ncRNA stability, delivery efficiency, valve-cell selectivity, and biosafety (160–172). However, these approaches remain largely preclinical in the context of AS. At present, there are no approved lncRNA-based therapies, completed clinical trials, or published clinical case reports demonstrating that direct RNA delivery can halt or reverse aortic valve calcification or treat the aortic valve lesion in AS. Thus, ncRNA-based strategies should currently be viewed as promising tools for biomarker discovery, disease phenotyping, and mechanistic target identification rather than clinically established therapies for AS. Clinical translation will require large-scale human validation, physiologically relevant disease models, rigorous preclinical safety profiling, and delivery platforms capable of selectively targeting relevant valve-cell populations within diseased human aortic valves.

## Conclusion and future directions

Looking ahead, ncRNAs hold a transformative potential for the diagnosis and treatment of AS and ADs. The continuing discovery of entirely new regulatory mechanisms involving circulating and tissue-resident ncRNAs offers renewed hope for innovative diagnostic and therapeutic strategies. As research advances, ncRNA-based therapeutics may open new avenues for precision medicine in CVDs, provided rigorous preclinical evaluation, robust safety profiling, and a comprehensive understanding of the complex regulatory networks in which these molecules operate underpin their translation. In addition, substantial biological, technical, and translational barriers may limit their clinical implementation. Key priorities for the field must include: (i) large-scale clinical validation of ncRNA biomarkers, (ii) refinement of delivery platforms for targeted therapeutics, (iii) development of combinatorial approaches targeting shared pathological pathways, and (iv) deeper investigation of the variability in ncRNA-mediated regulation across different CV pathologies and cell types due to their strong cellular context dependence. Some critical challenges include insufficient large-scale validation studies and strong databases for accurate interpretations of ncRNA expression patterns: non-coding RNA interactions with proteins, between similar and/or different molecules. Moreover, the context-dependent nature of ncRNAs, together with limited evolutionary conservation and lack of animal models, makes the interpretation and translational relevance difficult. Thus, ncRNA research has gained strong understanding in CVDs and AS pathogenesis and continues opening promising windows for diagnosis, disease stratification, and development of novel therapeutics, holding considerable potential to become an important component for future precision medicine strategies.
